# The Effects of Rebamipide 2% Ophthalmic Solution Application on Murine Subbasal Corneal Nerves After Environmental Dry Eye Stress

**DOI:** 10.3390/ijms20164031

**Published:** 2019-08-18

**Authors:** Cem Simsek, Takashi Kojima, Shigeru Nakamura, Murat Dogru, Kazuo Tsubota

**Affiliations:** 1 Department of Ophthalmology, Keio University School of Medicine, Tokyo 1608582, Japan; 2 Department of Ophthalmology, Mugla Sitki Kocman University School of Medicine, Mugla 48000, Turkey

**Keywords:** dry eye, rebamipide, corneal sensitivity, corneal subbasal nerves, in vivo confocal microscopy

## Abstract

Rebamipide ophthalmic solution is a mucin secretagogue which is an important therapeutic agent in the treatment of dry eye. It has been noted that dry eye in office workers is associated with a decrease in secretory mucin. This study aimed to evaluate the effects of 2% rebamipide ophthalmic solution in mice subjected to environmental dry eye stress (EDES), which mimics the conditions of office workers. Thirty eyes from thirty BALB/c mice (eight-week-old males) were divided into three treatment groups: artificial tear (vehicle), 2% rebamipide ophthalmic solution, and 0.1% hyaluronic acid (HA) ophthalmic solution. After four days of pretreatment, mice were exposed to EDES for three days. The corneal subbasal nerve and inflammatory cells were then examined using in vivo confocal microscopy. Following EDES exposure, the lissamine green staining score was significantly lower and corneal sensitivity was more preserved in the 2% rebamipide group than in the HA group. In addition, the subbasal nerve fiber density was significantly higher and the DC density was significantly lower in the 2% rebamipide group than in the HA group. Overall, the topical rebamipide ophthalmic solution showed more favorable therapeutic effects when compared to the HA ophthalmic solution in a mouse model of EDES, likely owing to its anti-inflammatory and neuroprotective effects.

## 1. Introduction

Prevalence of definite or probable dry eye in office workers was reported to be high at 60.2% for men and 76.5% for women, according to the study by Uchino et al [1]. It seems that office workers are affected by long-time visual display terminal (VDT) work, dry environment by air conditioning, and mental stress by sitting and working at the same place. We previously developed a rat swing model [2] that mimics the environment in office workers, and further refined it to develop an environmental dry eye stress model (EDES) [3].

The cornea is a highly-innervated tissue, which harbors 300 to 600 times more nerve terminals compared with the skin [4,5]. A significant portion of corneal innervation is supplied by the terminal portions of the ophthalmic and maxillary branches of the trigeminal nerve [6]. The 2017 International Dry Eye Workshop II (DEWS II) report stated that neurosensory abnormalities play an important role in the pathogenesis of dry eye disease (DED) [7]. Several studies have shown that the subbasal nerve maintains corneal sensation, and corneal epithelial integrity and continuity [5,8]. The number of nerves supplying epithelial proliferation, integrity, and viability were found to be decreased in various ocular surface and systemic diseases, leading to disorders of the ocular surface and decreased aqueous tear secretion [9,10].

Quantitative measurements of the corneal subbasal nerve density are frequently used to assess alterations in the corneal neural network [6]. Other parameters including tortuosity, reflectivity of nerve fibers, and dendritic cell (DC) density can also be investigated by in vivo confocal microscopy (IVCM), providing valuable information about DED [8]. In our previous work, we showed that a decreased corneal subbasal nerve density and increased DC numbers were detected after short-term exposure to environmental dry eye stress [3].

At present, tear film oriented therapy (TFOT) is the treatment strategy for dry eye disease proposed by the Asia Dry Eye Society [11], and involves treating the tear film according to the specific layer that is abnormal. Secretory mucin in the aqueous layer of the tear film functions in enhancing the retention of the tear on the ocular surface. The membrane-bound mucin changes the surface layer of the epithelium to be hydrophilic and maintains the wettability of the ocular surface. In dry eye disease in office workers, it has been reported that tear volume is normal and decreased tear stability is more prevalent [1]. It is also reported that the longer the VDT working time, the lower the concentration of MUC5AC in the tear [12]. These studies indicate that secretory mucin plays a major role in the pathogenesis of dry eye disease in office workers.

Rebamipide is a quinolinone derivative with mucin producing activity, which was originally developed as a mucosal protective agent for the treatment of peptic ulcers and chronic gastritis. Rebamipide ophthalmic solution (Mucosta®; Otsuka Pharmaceutical, Chiyoda, Japan), a mucin secretagogue, has been approved in Japan and is commonly used to treat the mucin layer according to the aforementioned TFOT concept. Rebamipide elevates the expression levels of MUC1, MUC4, and MUC16 gene via the activation of epidermal growth factor receptor and increases mucin-like glycoprotein production in human corneal epithelial cells [13,14]. Previous studies have shown that topical application of rebamipide significantly improves symptoms, corneal fluorescein staining, conjunctival lissamine green staining, and tear film break-up time (TBUT) in DED [15,16,17].

In vivo confocal microscopy is a novel non-invasive imaging method that enables corneal evaluation at the cellular level and is frequently used in the differential diagnosis and follow-up of several diseases [18]. High resolution images of corneal subbasal nerves and immune cells are obtained by IVCM [18]. Several studies evaluating healthy and pathological corneal subbasal nerves have been published coinciding with the increased clinical use of IVCM [18,19].

Although the effects of rebamipide eye drops on the ocular surface epithelium, tear function, and the conjunctiva have been investigated previously [14,15,20,21,22], its effects on corneal subbasal nerves have not yet been examined. Moreover, there is a paucity of studies which have evaluated rebamipide eye drops in VDT animal models. This study aimed to compare the effects of 2% rebamipide eye drops with 0.1% sodium hyaluronate (HA) eye drops on tear function, ocular surface abnormalities, corneal sensation, and subbasal corneal nerves in male BALB/c mice after exposure to EDES by IVCM. In the clinical settings, office workers with dry eye are instructed to perform eye drop treatments not only before but during work as well. Therefore, this study was designed to perform topical treatment not only during exposure to EDES but also before EDES, and the treatment effect of the eye drops was evaluated. In our study, 0.1% HA was selected as a control as it is commonly used in Japan and its therapeutic effect on DED has been reported by many studies [23,24].

## 2. Results

### 2.1. Aqueous Tear Secretion Quantity and Changes in Tear Film Stability

**Figure 1:**
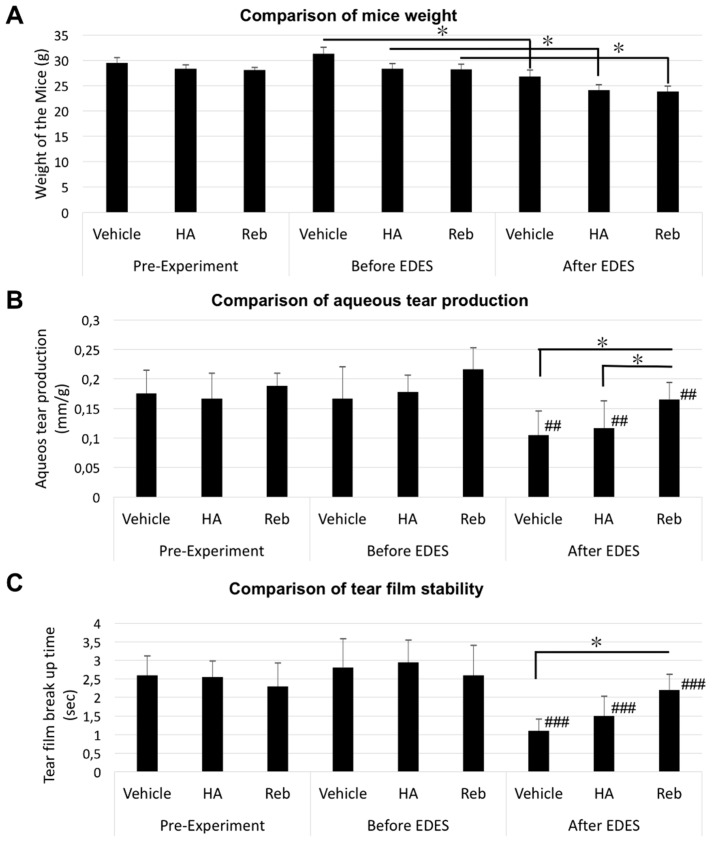
Weight of the mice, aqueous tear production and tear film stability measurement. (**A**) The mean weight decreased significantly in all groups after exposure to EDES. (**B**) A marked decrease in aqueous tear quantity can be seen in all groups after EDES exposure for three days (*p* < 0.0001). After EDES exposure, aqueous tear quantity in the rebamipide group (Reb) was significantly higher than that in the vehicle (*p* = 0.013) and HA groups (*p* = 0.022). (**C**) The mean TBUT decreased significantly with EDES exposure in all groups (# *p* < 0.0001). Following EDES exposure, the mean TBUT in the rebamipide group was significantly longer than that in the vehicle group (*p* = 0.0019). # represents comparison between time points (# *p* < 0.05, ## *p* < 0.001, ### *p* < 0.0001) and * represents comparison between treatment groups (* *p* < 0.05, ** *p* < 0.001, *** *p* < 0.0001).

We evaluated the alterations in body weights pre-experiment, after four days of eye drop instillation/before EDES, and three days following EDES exposure. We demonstrated that three days of EDES exposure caused significant weight loss in mice (*p* < 0.0001, Figure 1A).

We assessed weight-adjusted aqueous tear production quantity using the Zone-Quick cotton thread test. The analysis of variance (ANOVA) revealed significant differences both between time points (*p* < 0.0001) and treatment groups (*p* = 0.0013). The multiple comparisons test showed that tear volume after exposure to EDES in all groups was significantly lower than that before exposure to EDES (all groups: *p* < 0.001). After exposure to EDES, the mean aqueous tear production values in the rebamipide group (0.165 ± 0.029 mm/g) was significantly higher than those in the vehicle (0.105 ± 0.041 mm/g, *p* = 0.013) and HA groups (0.117 ± 0.046 mm/g, *p* = 0.022; Figure 1B).

In relation to tear film stability, the ANOVA analysis revealed significant differences both between time points (*p* < 0.0001) and treatment groups (*p* = 0.021). The multiple comparisons test revealed that TBUT after exposure to EDES in all groups was significantly lower than that before exposure to EDES (all groups: *p* < 0.0001). After exposure to EDES, the mean TBUT values in the rebamipide group (2.20 ± 0.4 sec) were significantly higher than those in the vehicle group (1.10 ± 0.32 sec, *p* = 0.0064), but negligibly different from those in the HA group (1.50 ± 0.53 mm/g, *p* = 0.087; Figure 1C). All measured values are shown in the supplemental material.

### 2.2. Changes in Vital Staining Score

**Figure 2:**
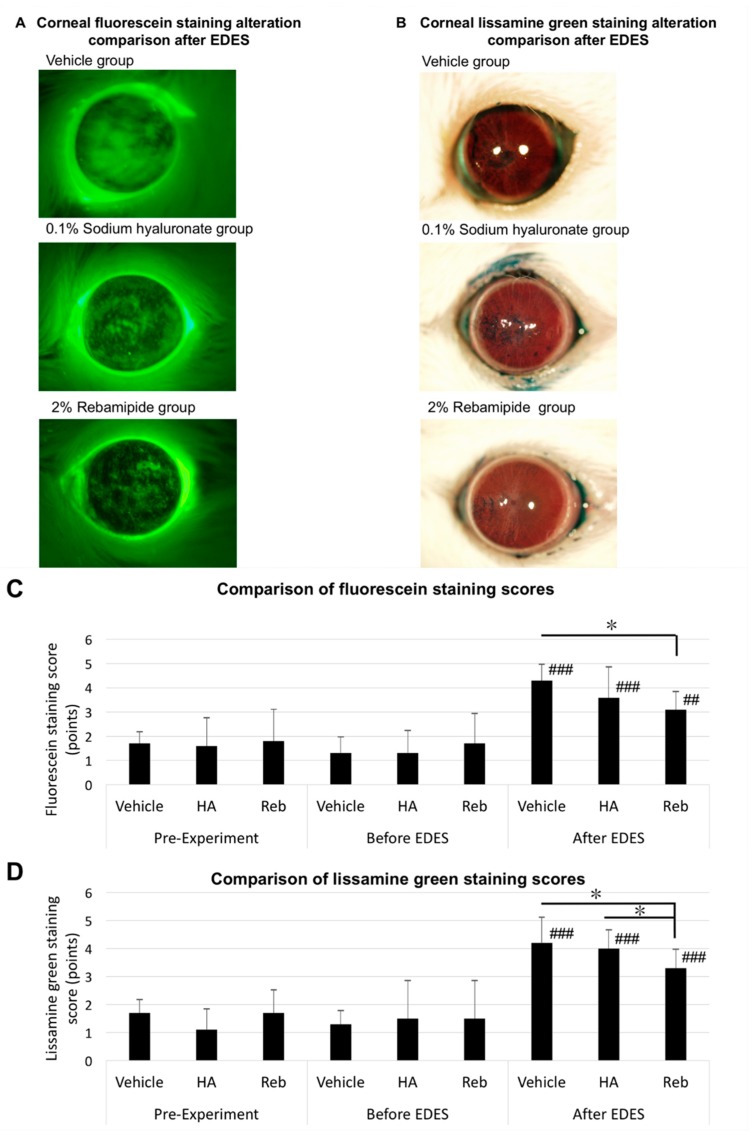
Representative anterior segment photographs and comparison of corneal fluorescein and lissamine green staining scores in 0.1% HA and 2% rebamipide instillation groups. Representative post-EDES fluorescein (**A**) and lissamine green (**B**) staining photographs show denser staining in the vehicle than HA and rebamipide groups. (**C**) The mean fluorescein staining score in all groups significantly increased after EDES exposure (*p* < 0.0001). Please note a significant impairment of the fluorescein staining scores in the vehicle group compared to the rebamipide group after EDES exposure (*p* = 0.006). (**D**) The mean lissamine green staining score in all groups significantly increased after EDES exposure (*p* < 0.0001). Please note a significant impairment of lissamine green staining scores in the vehicle (*p* = 0.007) and HA groups (*p* = 0.025) compared to the rebamipide group after EDES exposure. # represents comparison between time points (# *p* < 0.05, ## *p* < 0.001, ### *p* < 0.0001) and * represents comparison between treatment groups (* *p* < 0.05, ** *p* < 0.001, *** *p* < 0.0001).

We evaluated the changes in corneal fluorescein and lissamine green staining scores pre-experiment, after four days of eye drop instillation/before EDES, and three days following EDES exposure. The ANOVA analysis revealed significant differences both between time points (fluorescein and lissamine green, *p* < 0.0001) and treatment groups (fluorescein, *p* = 0.016; lissamine green, *p* = 0.019). The multiple comparisons test revealed that the fluorescein staining score after exposure to EDES in all groups was significantly higher than that before exposure to EDES (vehicle and HA: *p* < 0.0001, rebamipide: *p* < 0.001). After exposure to EDES, the mean fluorescein staining score in the rebamipide group (4.30 ± 0.67 points) was significantly lower than that in the vehicle group (3.10 ± 0.74 points, *p* = 0.006), but negligibly different from that in the HA group (3.60 ± 1.26 points, *p* = 0.18; Figure 2A). Similarly, the multiple comparisons test revealed that the lissamine green staining score after exposure to EDES in all groups was significantly higher than that before exposure to EDES (all groups: *p* < 0.0001). After exposure to EDES, the mean lissamine green staining score in the rebamipide group (3.30 ± 0.67 points) was significantly lower than that in the vehicle group (4.20 ± 0.62 points, *p* = 0.007) and the HA group (4.00 ± 0.67 points, *p* = 0.025; Figure 2B).

### 2.3. Corneal Sensitivity Alterations

**Figure 3:**
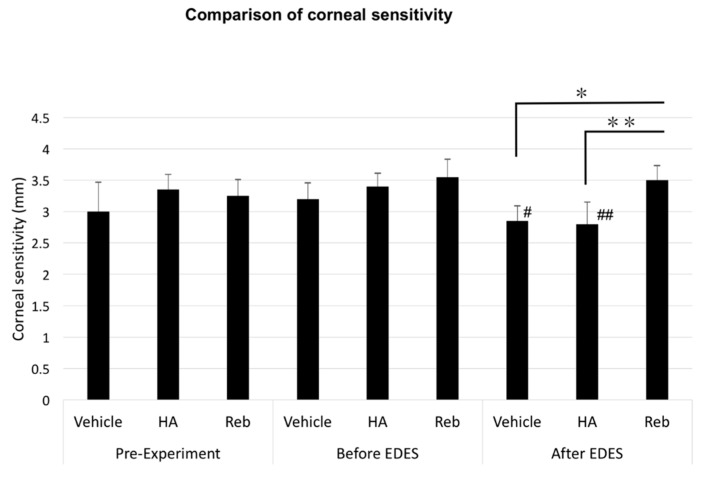
Quantitative analysis of corneal sensitivity. Please note the significant decrease in corneal sensitivity in the vehicle (*p* = 0.014) and HA groups (*p* = 0.0009) after EDES exposure. After exposure to EDES, corneal sensitivity in the rebamipide group (Reb) was significantly higher than that in the vehicle (*p* = 0.003) and HA groups (*p* = 0.007). # represents comparison between time points (# *p* < 0.05, ## *p* < 0.001, ### *p* < 0.0001) and * represents comparison between treatment groups (* *p* < 0.05, ** *p* < 0.001, *** *p* < 0.0001).

There was a significant difference between time points and treatment groups (time points, *p* = 0.0003; treatment groups, *p* < 0.0001) with regards to changes in corneal sensitivity. The corneal sensitivity in vehicle and HA groups significantly decreased after exposure to EDES (*p* < 0.05 and *p* < 0.001, respectively). The multiple comparisons test revealed that corneal sensitivity values after exposure to EDES in vehicle and HA groups were significantly lower than that before exposure to EDES (*p* = 0.014 and *p* = 0.0009, respectively). After exposure to EDES, the mean corneal sensitivity value in the rebamipide group (3.50 ± 0.24 sec) was significantly higher than that in the vehicle group (2.85 ± 0.24 sec, *p* = 0.003) and the HA group (2.80 ± 0.35 mm/g, *p* = 0.007; Figure 3).

### 2.4. In Vivo Confocal Microscopy Findings

**Figure 4:**
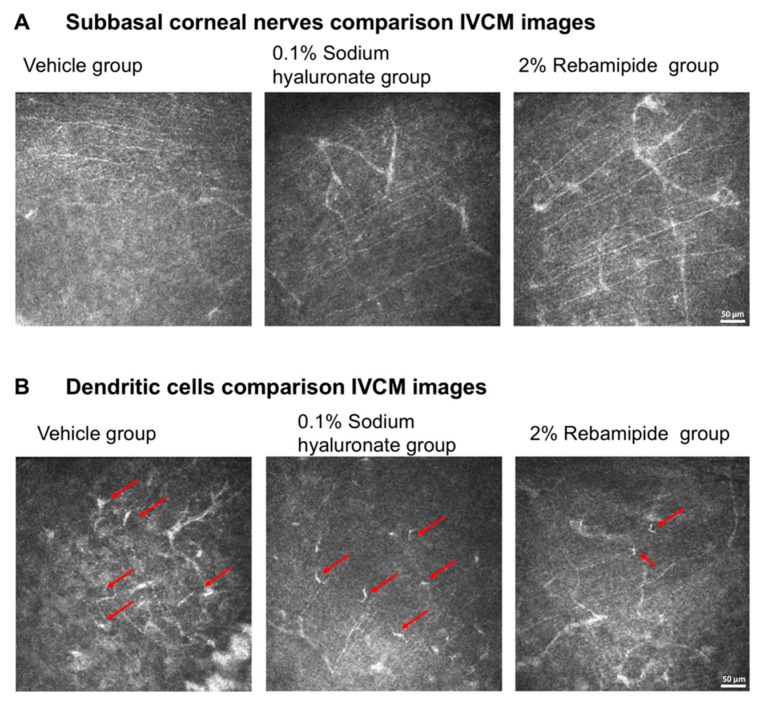
Representative in vivo confocal microscopy images in the vehicle, HA, and rebamipide treatment groups. Representative IVCM images of mice after exposure to EDES three days. (**A**) A significant reduction in corneal subbasal nerve fibers can be seen clearly in the vehicle and HA groups. (**B**) A significantly lower DC density in the rebamipide group was found compared with the vehicle and HA groups after exposure to EDES for three days. Red arrows indicate the DCs. Scale bar: 50 µm.

A total of 240 images including 3474 nerves were examined for density of nerve fibers (NFD) by two examiners (Figure 4).

**Figure 5:**
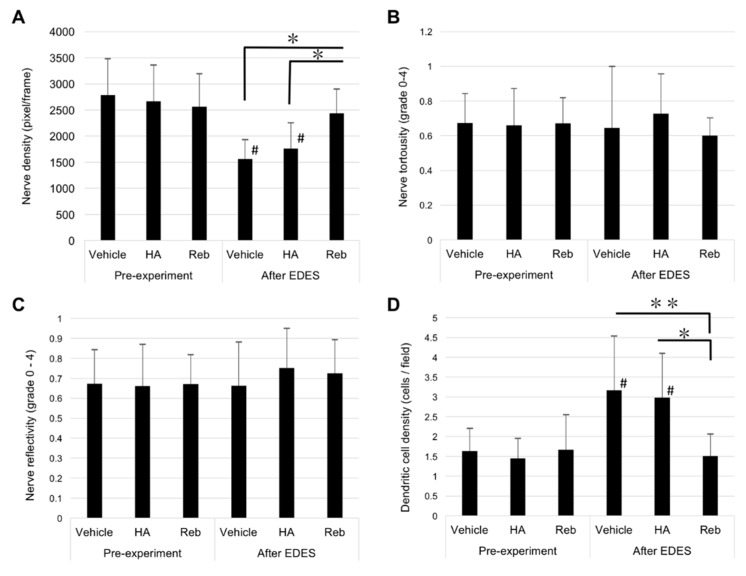
Quantitative analysis of in vivo confocal microscopy images for nerve density, tortuosity, reflectivity, and DC density. (**A**) A significant reduction of nerve density was observed in the vehicle and HA groups after exposure to EDES (both groups; *p* < 0.0001). The corneal nerve density in the rebamipide group (Reb) was significantly higher than the vehicle (*p* = 0.004) and HA groups (*p* = 0.007). (**B**) There were no significant differences in nerve tortuosity between time points (*p* = 0.944) or treatments (*p* = 0.759). (**C**) There were also no significant differences in nerve reflectivity between time points (*p* = 0.539) and treatments (*p* = 0.903). (**D**) The DC density in the vehicle and HA groups significantly increased after exposure to EDES (vehicle group, *p* = 0.002; HA group, *p* = 0.004). The DC density in the rebamipide group was lower than the vehicle (*p* = 0.0004) and HA (* *p* = 0.006) groups. # represents comparison between time points (# *p* < 0.05, ## *p* < 0.001, ### *p* < 0.0001) and * represents comparison between treatment groups (* *p* < 0.05, ** *p* < 0.001, *** *p* < 0.0001).

The mean NFD was 2788 ± 691 pixels/frame in the vehicle group, 2668 ± 689 pixels/frame in the HA group, and 2562 ± 629 pixels/frame in the rebamipide group before exposure to EDES. After exposure to EDES three days, the mean NFD was 1504 ± 347 pixels/frame in the vehicle group, 1669 ± 412 pixels/frame in the HA group, and 2331 ± 360 pixels/frame in the rebamipide group. The ANOVA analysis revealed that there were significant differences both between time points and between treatment groups (*p* = 0.0002 and *p* < 0.0001, respectively). The mean NFD in vehicle and HA groups significantly decreased after exposure to EDES (*p* = 0.004 and *p* = 0.007, respectively). Moreover, the mean NFD in the rebamipide group was significantly greater than the vehicle and HA groups (*p* < 0.0001 and *p* = 0.003, respectively; Figure 5A).

We also examined the tortuosity and reflectivity of 1440 nerves from 480 images using our previously defined grading scale [3]. After exposure to EDES, the mean subbasal nerve tortuosity grades in the vehicle, HA, and rebamipide groups were 0.66 ± 0.22, 0.73 ± 0.23, and 0.60 ± 0.10, respectively. There was no significant difference in the tortuosity values between the three treatment groups or two time points (*p* = 0.759 and *p* = 0.944, respectively; Figure 5B).

The mean subbasal corneal nerve reflectivity grades in the vehicle, HA, and rebamipide groups were 0.65 ± 0.36, 0.75 ± 0.20, and 0.72 ± 0.17, respectively. There was no difference between the three treatment groups or two time points (*p* = 0.903 and *p* = 0.539, respectively; Figure 5C).

The ANOVA analysis revealed that DC density was significantly different between time points (*p* = 0.0007) and treatment groups (*p* = 0.0361). The mean subbasal DC densities in the vehicle, HA, and rebamipide groups were 3.17 ± 1.37, 2.98 ± 1.12, and 1.51 ± 0.56 cells/field, respectively. The multiple comparisons test revealed that the DC density in the rebamipide group was significantly lower than that in the vehicle and HA groups after exposure to EDES (rebamipide vs vehicle, *p* = 0.0004; rebamipide vs HA, *p* = 0.006; Figure 5D).

## 3. Discussion

We have demonstrated that the four-day application of the eye drops used in the study did not alter the TBUT in any of the groups until EDES introduction. However, after exposure to EDES, the rebamipide-treated group exhibited significantly more stable tear film compared to the vehicle group. A previous clinical study has shown that short-term rebamipide instillation has protective and therapeutic effects on corneal staining scores [25]. The same study also showed that administration of rebamipide ophthalmic solution for two weeks resulted in a significant improvement of lissamine green staining score compared with HA eye drops [25]. Previous human and animal studies demonstrated that rebamipide increases the number of periodic acid-Schiff-positive goblet cells and mucin levels in the conjunctiva [22,26].

We found that corneal staining scores did not change in any of the groups after four days of pretreatment. At the outset of the pre-experiment stage, all groups showed worsening of fluorescein staining scores. However, the mean fluorescein staining score in the rebamipide group was better than that in the vehicle and HA groups. We have previously shown that topical administration of rebamipide in an aging mouse model of DED (*Sod1* knockout mice) mitigated corneal epithelial damage by promoting tear stability and increasing the expression of MUC5AC mRNA after two weeks of treatment [21]. Furthermore, it had a therapeutic effect on conjunctival epithelial keratinization after a relatively short period of treatment [22]. We speculate that these mechanisms may also apply to the current mouse model of EDES.

In this study, the effects of rebamipide and HA eye drops on the corneal subbasal nerves (CSN) in mice exposed to EDES were primarily examined by comparing the NFD levels. Both vehicle and HA groups, but not the rebamipide group, showed significant decreases after exposure to EDES. Moreover, the rebamipide group showed significantly higher NFDs compared with the vehicle and HA groups after exposure to EDES. We hypothesize that rebamipide eye drops have a neuroprotective effect on CSN.

There are many studies utilizing IVCM to evaluate qualitative and quantitative variations in corneal nerves due to DED [27,28] and have typically focused on the corneal subbasal nerve density. Although a majority of studies report a decrease in the corneal nerve density [29,30,31,32], there are also studies reporting an increase in nerve density in patients with Sjögren’s syndrome [33]. Studies by Hosal et al. [34] and Tuominen et al. [35], however, showed no changes in corneal superficial nerve density between DED patients and controls. The variable results obtained by different studies are thought to be due to differences in paraplegia levels, allodynia levels, inflammation levels, differences in the corneal nerve damage severity, differences in stages of DED, and differences in neural regeneration/degenerative patterns. Based on these studies, our EDES-exposed mouse models and IVCM observation are essential for evaluating the pathophysiology of DED.

Other morphological parameters that have been commonly evaluated with CSN include tortuosity, reflexivity, budding patterns, and DC density [8,29,30,33,35,36]. Previous studies have shown that the aforementioned parameters are worse in dry eye patients, possibly due to neural regeneration after damage to the subbasal corneal nerves. In patients with DED, an increase in the intensity of DCs and other immunocytes was also detected using IVCM [27,29,30,33,37] and could be correlated with their clinical symptoms [38]. Therefore, IVCM is considered a significant diagnostic method that can aid in the diagnosis and treatment of DED. In this study, we compared the effect of rebamipide eye drops with vehicle and HA eye drops on DC density after a seven-day experimental procedure. We demonstrated a notable reduction in DC density with topical rebamipide eye drop instillation compared to vehicle and HA eye drop instillations. A previous study by Arakaki et al., (2014) showed that topical rebamipide application decreased inflammation in ocular autoimmune lesions in a murine model of Sjögren’s syndrome and had a protective effect on the ocular surface [39]. Additionally, Ueta et al., (2013) demonstrated that the topical instillation of rebamipide on the ocular surface may reduce ocular surface inflammation by decreasing the release of cytokines by the epithelial cells in this region, which is compatible with the results of our study [40]. It remains unclear how ocular surface inflammation affects the subbasal nerve changes in our mouse model. Local inflammation and peripheral nerve damage can occur due to stress in the ocular mucosal epithelium in association with reduced tear production and increased osmolarity [41]. Moreover, local inflammation and nerve damage could cause short- and long-term genetic and molecular alterations in the primary sensory neurons [42]. On the other hand, a previous study has suggested that corneal subbasal nerves have a potential role in neuroprotection [43].

Normal corneal sensitivity is essential to maintain a healthy ocular surface. We found decreased corneal sensitivity in the vehicle and HA groups but not in the rebamipide group after exposure to EDES. These results were consistent with the results of CSN density. Although the detailed mechanism is still unknown, these results suggest that rebamipide treatment may contribute to the maintenance of CSN and corneal sensitivity due to increased tear stability as a result of mucin production and the inhibition of ocular surface inflammation.

Tear secretion in all groups decreased after exposure to EDES, although, interestingly, the rebamipide group exhibited a higher value than did the vehicle and HA groups. From these results, we speculate that the neuroprotective function of rebamipide treatment preserved tear secretion after exposure to EDES. Another explanation may be an improvement in ocular surface wettability by the increased expression of membrane-type mucin. Previous reports have also shown a decrease in corneal sensitivity in mouse dry eye models [44,45], although to a much greater extent than that observed in our study. Given that the examination method (material and diameter of filament) and dry eye models used were different, these results cannot be directly compared.

In the current study, we also assessed the grades of tortuosity and reflexivity, and did not detect any significant changes in these parameters. We presume that this was due to the lack of time for regeneration of corneal nerves after environmental stress over the three-day period. We believe that it may be possible to obtain variations in these parameters by performing longer term studies on mice in the future.

In a double-blind placebo-controlled study, rebamipide was shown to improve symptom score more than placebo treatment in functional dyspepsia [46]. It has been reported that, in addition to mucin secretion, the pharmacological mechanism of rebamipide includes anti-oxidative and anti-inflammatory actions [47]. However, the detailed molecular mechanism underlying its activity remains unknown. In a rat model of osteoarthritis, rebamipide has been reported to suppress pain [48]. In this report, rebamipide decreased the inflammatory mediators matrix metaproteinase-13, interleukin-1β, and hypoxia-inducible factor-2α, and the oxidative stress mediators inducible nitric oxide synthetase and nitrotyrosine within the knee joint. It has recently been noted that symptoms in dry eye may involve an underlying neuropathic mechanism [49]. Since the rebamipide ophthalmic solution exhibited a neuroprotective action, the previously reported alleviation of dry eye symptoms may be due to anti-inflammatory or neuroprotective actions via mucin secretion.

Our study has several limitations, including the exclusive use of male mice and the fact that we restricted our investigations to the acute phase of nerve changes after dry eye stress. Given that dry eye is a chronic disease, it would be interesting to further investigate whether rebamipide would be effective for chronic dry eye changes in corneal nerves. Furthermore, we did not take note of nerve healing after exposure to EDES. We designed the current study to investigate both the preventive and treatment effects of rebamipide instillation together. However, to evaluate the distinct effects of rebamipide on prevention and treatment, future work will focus on assessing the effect of rebamipide eye drops after the initiation of dry eye disease.

In our study, we chose to use BALB/c mice as they are known to have a higher density of subepithelial nerves than are C57BL/6 mice, as observed by IVCM. Although BALB/c mice are a Th2-prone strain, human dry eye is more associated with Th1 and Th17 than with Th2. Further evaluation of the morphological changes seen in different mice strains, such as C57BL/6 mice, need to be carried out in the future.

As aforementioned in this study, eye drops were used prior to exposure to environmental dry eye stress to simulate the actual office worker’s dry eye treatment. There were no significant differences in tear function and vital stain scores before and after pretreatment. However, investigation of whether pretreatment or treatment under EDES affects the changes in corneal nerve and tear functions is essential. In the future, it will be necessary to examine in detail whether pretreatment or treatment under EDES has a greater impact on the ocular surface health in the EDES model mice.

## 4. Materials and Methods

### 4.1. Animals and Experimental Procedure

**Figure 6:**
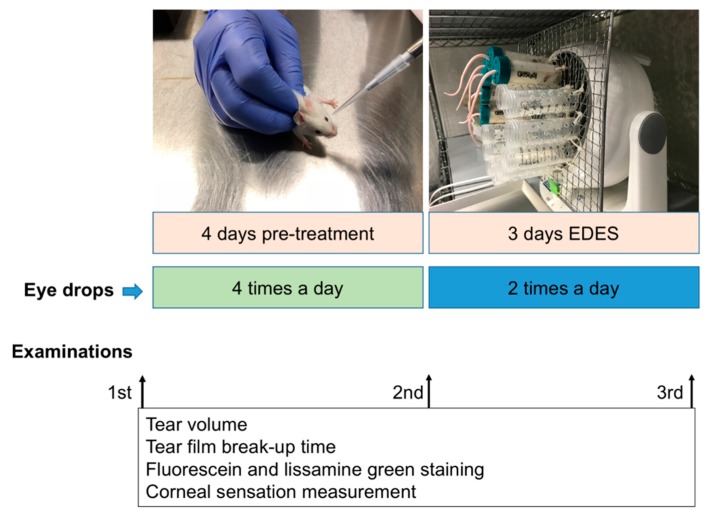
Flowchart of the experimental procedure. A flowchart of the whole experimental procedure along with representative photographs of eye drop instillation and the mouse model of EDES are shown.

Eight-week-old BALB/c mice were obtained from CLEA Japan (Yokohama, Japan) and used to investigate alterations in the ocular surface and CSN after application of 2% rebamipide or preservative-free 0.1% HA eye drops (0.1% Hyalein, Santen Pharmaceutical, Osaka, Japan). Thirty right eyes from 30 male BALB/c mice (*n* = 30) were included in this study. Sample size calculations were carried out using the G^*^Power software (Heinrich Heine, Düsseldorf University, Düsseldorf, Germany) by specifying input parameters including the standard deviation, effect size, and type 1 error [50]. All mice were first divided into three groups of 10 each; the vehicle group treated with preservative-free artificial tear eye drops (Soft Santear, Santen, Osaka, Japan), the rebamipide group treated with 2% rebamipide eye drops, and the HA group treated with 0.1% HA eye drops four times daily for four days (applied at 9:00 am, 12:00 pm, 3:00 pm, and 6:00 pm; Figure 6). The mice were then exposed to EDES for three days. During this period, eye drops were instilled twice daily, before and after exposure to EDES (Figure 6).

All studies were performed in accordance with the Association for Research in Vision and Ophthalmology (ARVO) Statement for the Use of Animals in Ophthalmic and Vision Research. The Animal Experimentation Ethics Committee of the Keio University School of Medicine approved the current research procedures (08067-7, 25 October 2017).

### 4.2. Application of Environmental Dry Eye Stress

All mice were exposed to EDES for five hours per day (9:00 am to 2:00 pm) for three days. The implementation of EDES is described in detail in our previous study [3]. Briefly, the mice were isolated in singular equal-sized small compartments and exposed to a stable, continuous airflow (4 m/s) produced by an 18-cm-diameter electric fan. The fan was set 5 cm away from the mice, mean relative humidity in the room was set to 25 ± 5%, and mean room temperature was fixed at 23 ± 2°C.

### 4.3. Aqueous Tear Secretion Quantity and Tear Film Stability Assessment

Aqueous tear secretion quantity and tear film stability were examined at three different time points: pre-experiment, before exposure to EDES, and after exposure to EDES for each mouse (Figure 6). Aqueous tear secretion quantity and the TBUT measurements were performed according to protocols described in our previous study [3]. Briefly, aqueous tear secretion quantity was evaluated by using phenol red-treated cotton threads (Zone-Quick, Showa Yakuhin Kako Co., Ltd., Tokyo, Japan) on animals without anesthesia. We placed the cotton threads into the lateral canthus using micro forceps for 30 seconds. The wet length of the cotton threads was measured in millimeters using a ruler provided by the manufacturer. The weight-adjusted tear volume was then calculated by dividing the wet length by the body weight of the animal at the time of measurement.

The TBUT was examined to assess the ocular surface tear film stability. First, one drop containing 1 μL of 2% sterile fluorescein was applied onto the ocular surface using a micropipette and excess fluorescein was wiped from the lateral canthus. After a natural blink response was stimulated by an air puff produced by a 1-mL syringe, the TBUT was examined three times with portable slit-lamp biomicroscopy using cobalt blue light (Kowa Co., Ltd., Tokyo, Japan) and the mean of these results was then calculated.

### 4.4. Ocular Surface Vital Staining Assessment

All mice underwent ocular vital staining assessments before the experiment and before and after exposure to EDES (Figure 6). Fluorescein staining was examined 2 min after TBUT assessment using a scoring system previously described by Shimmura et al. [51]. The cornea was divided into three parts (superior, middle, and inferior) and each part was scored between 0 and 3 points according to the extent of the area stained. A score of 0 signified no staining, 1 signified mild staining (less than one-third of the area), 2 signified moderate staining (less than two-thirds of the area), and 3 signified severe staining (more than two-thirds of the area) for a minimum and maximum total score of 0 and 9 points, respectively). Five microliters of phosphate-buffered saline (PBS) was used to wash out fluorescein, which was removed from the lateral canthus before repeating the same procedure with the lissamine green dye. Vital staining photographs were recorded in JPEG format using a microscope connected to a digital camera with the same settings maintained for all mice.

### 4.5. Corneal Sensitivity Assessment

We employed a modified Cochet-Bonnet esthesiometer as reported in previous studies [44,45]. Hi Loop 0.3 nylon filaments (Unitika Ltd. Tokyo, Japan), with 0.09 mm diameter, were used to evaluate the corneal sensitivity of mice. Seven filaments ranging from 0.5 cm to 4 cm in length were prepared by attaching them to a holder. Starting from the longest filament (4 cm, the lowest pressure), each length of nylon filament was applied three times to stimulate the corneal nerves and cause a corneal blink response. The filament contacted the central cornea perpendicularly while avoiding the eyelashes and lid margins. The nylon filament length was reduced by 0.5-cm increments until the mouse demonstrated a complete blink reflex, which was defined as two out of three applications yielding positive blink responses. This procedure was performed at the central cornea of both eyes under a magnifying glass and without any light sources. Three measurements were recorded at each time point and the average value was taken for analysis. The reproducibility of this method in obtaining corneal sensitivity measurements was confirmed prior to conducting all experiments.

### 4.6. In Vivo Laser Scanning Confocal Microscopic Examination and Image Analysis

We performed ocular surface analyses using IVCM with an anterior segment adaptor. The Rostock Cornea Module of the Heidelberg Retina Tomograph II (HRT; Heidelberg Engineering, Heidelberg, Germany) was used to examine corneal morphological alterations. Following intraperitoneal administration of anesthesia (4 mg/mL of xylazine and 6 mg/mL of ketamine), a carbomer 2% gel (Comfort gel, Berlin, Germany) was applied to the cornea to avoid exposure and dryness during IVCM examination. The diode laser source, internally mounted in the HRT, supplied a 670 nm red light. High resolution real-time images obtained by IVCM consisted of 384 × 384 pixels covering an area of 400 × 400 μm (horizontal x vertical) with a lateral resolution of 1 µm/pixel. Starting the scans from the corneal epithelial side, the subepithelial nerve plexus was observed at scan depths of 40 to 50 μm from the epithelial side in all mice where the images were recorded as a JPEG frame with 8-bit resolution and a 128-bit binary floating-point format. Six to eight complete sequences, each containing 100 images, were recorded from each cornea (each frame representing an area of 160 µm^2^) with the approximate duration of IVCM examination being eight minutes per eye. Four non-overlapping representative images of each cornea were selected for corneal morphological analysis. As previously described [3], two masked experienced researchers analyzed in-focus images for corneal nerve morphological features and investigated the NFD, tortuosity and reflectivity of CSN, and DC density as follows:

(1) The NFD was assessed by measuring the total length of CSN fibers within a frame (160,000 µm^2^), as defined in our previous study [3]. After semi-automatically marking the CSN in each frame, the NFD was automatically measured by the NeuronJ plug-in for ImageJ software (National Institutes of Health, Bethesda, MD, USA). Four different representative images for each cornea were analyzed in pixels. The mean CSN for each cornea was calculated by averaging these total values. The data were determined as density (µm/mm^2^) ± standard error of the mean (SEM).
(2) Nerve tortuosity was evaluated at the subbasal layer according to previously published grading scales [3]. The grading scales consisted of a series of images derived from our previous work and other studies of corneal innervation using confocal microscopy. Briefly, Oliveira-Soto and Efron (2001) classified human corneal nerve tortuosity into five grades [52]. Similarly, in our previous study, we classified mice corneal nerve tortuosity into five grades, ranging from 0 to 5, according to the subbasal nerve curve magnitude [3].
(3) The grading of nerve reflectivity was again performed according to our previous study [3]. In brief, we classified mice corneal nerve reflectivity into five grades from 0 to 5. Similarly, only nerves longer than 50% of the width frame underwent reflectivity assessment.
(4) The images were also examined for the density of DCs. The measurement of density of epithelial DCs was performed at the CSN plexus area and basal epithelial layer. Four representative images were used to evaluate the density of epithelial DCs.

### 4.7. Statistical Analysis

To assess the effects of two factors, i.e., time points and treatment groups, a two-way repeated measures ANOVA was performed to analyze tear quantity, vital staining scores, corneal sensation, and CSN parameters at different time points in each eye drop treatment group. Tukey’s test was then performed as a multiple comparisons test (post-hoc test). A p-value less than 0.05 was considered statistically significant.

## 5. Conclusions

Application of rebamipide ophthalmic solution appears to reduce inflammation at the corneal subbasal area and has neuroprotective effects on corneal nerves concomitant with maintaining tear secretion, compared with vehicle and HA ophthalmic solutions, in a mouse model of EDES.

## Supplementary Materials

Supplementary materials can be found at https://www.mdpi.com/1422-0067/20/16/4031/s1.

## References

[1] Uchino M., Yokoi N., Uchino Y., Dogru M., Kawashima M., Komuro A., Sonomura Y., Kato H., Kinoshita S., Schaumberg D.A. (2013). Prevalence of dry eye disease and its risk factors in visual display terminal users: The Osaka study. Am. J. Ophthalmol..

[2] Nakamura S., Kinoshita S., Yokoi N., Ogawa Y., Shibuya M., Nakashima H., Hisamura R., Imada T., Imagawa T., Uehara M. (2010). Lacrimal hypofunction as a new mechanism of dry eye in visual display terminal users. PLoS ONE.

[3] Simsek C., Kojima T., Dogru M., Tsubota K. (2018). Alterations of Murine Subbasal Corneal Nerves After Environmental Dry Eye Stress. Invest. Ophthalmol. Vis. Sci..

[4] Marfurt C.F., Cox J., Deek S., Dvorscak L. (2010). Anatomy of the human corneal innervation. Exp. Eye Res..

[5] Muller L.J., Marfurt C.F., Kruse F., Tervo T.M. (2003). Corneal nerves: structure, contents and function. Exp. Eye Res..

[6] Muller L.J., Pels L., Vrensen G.F. (1996). Ultrastructural organization of human corneal nerves. Invest. Ophthalmol. Vis. Sci..

[7] Craig J.P., Nelson J.D., Azar D.T., Belmonte C., Bron A.J., Chauhan S.K., de Paiva C.S., Gomes J.A.P., Hammitt K.M., Jones L. (2017). TFOS DEWS II Report Executive Summary. Ocul. Surf..

[8] Benitez del Castillo J.M., Wasfy M.A., Fernandez C., Garcia-Sanchez J. (2004). An in vivo confocal masked study on corneal epithelium and subbasal nerves in patients with dry eye. Invest. Ophthalmol. Vis. Sci..

[9] Davis E.A., Dohlman C.H. (2001). Neurotrophic keratitis. Int. Ophthalmol. Clin..

[10] Bonini S., Rama P., Olzi D., Lambiase A. (2003). Neurotrophic keratitis. Eye.

[11] Tsubota K., Yokoi N., Shimazaki J., Watanabe H., Dogru M., Yamada M., Kinoshita S., Kim H.M., Tchah H.W., Hyon J.Y. (2017). New Perspectives on Dry Eye Definition and Diagnosis: A Consensus Report by the Asia Dry Eye Society. Ocul. Surf..

[12] Uchino Y., Uchino M., Yokoi N., Dogru M., Kawashima M., Okada N., Inaba T., Tamaki S., Komuro A., Sonomura Y. (2014). Alteration of tear mucin 5AC in office workers using visual display terminals: The Osaka Study. JAMA Ophthalmol..

[13] Miri A., Alomar T., Nubile M., Al-Aqaba M., Lanzini M., Fares U., Said D.G., Lowe J., Dua H.S. (2012). In vivo confocal microscopic findings in patients with limbal stem cell deficiency. Br. J. Ophthalmol..

[14] Itoh S., Itoh K., Shinohara H. (2014). Regulation of human corneal epithelial mucins by rebamipide. Curr. Eye Res..

[15] Kinoshita S., Awamura S., Nakamichi N., Suzuki H., Oshiden K., Yokoi N., Rebamipide Ophthalmic Suspension Long-term Study Group (2014). A multicenter, open-label, 52-week study of 2% rebamipide (OPC-12759) ophthalmic suspension in patients with dry eye. Am. J. Ophthalmol..

[16] Kinoshita S., Awamura S., Oshiden K., Nakamichi N., Suzuki H., Yokoi N., Rebamipide Ophthalmic Suspension Phase II Study Group (2012). Rebamipide (OPC-12759) in the treatment of dry eye: A randomized, double-masked, multicenter, placebo-controlled phase II study. Ophthalmology.

[17] Simsek C., Dogru M., Shinzawa M., Den S., Kojima T., Iseda H., Suzuki M., Shibasaki Y., Yoshida N., Shimazaki J. (2019). The Efficacy of 2% Topical Rebamipide on Conjunctival Squamous Metaplasia and Goblet Cell Density in Dry Eye Disease. J. Ocul. Pharmacol. Ther..

[18] Cruzat A., Pavan-Langston D., Hamrah P. (2010). In vivo confocal microscopy of corneal nerves: analysis and clinical correlation. Semin Ophthalmol..

[19] Patel D.V., McGhee C.N. (2009). In vivo confocal microscopy of human corneal nerves in health, in ocular and systemic disease, and following corneal surgery: A review. Br. J. Ophthalmol..

[20] Itakura H., Kashima T., Itakura M., Akiyama H., Kishi S. (2013). Topical rebamipide improves lid wiper epitheliopathy. Clin Ophthalmol..

[21] Ohguchi T., Kojima T., Ibrahim O.M., Nagata T., Shimizu T., Shirasawa T., Kawakita T., Satake Y., Tsubota K., Shimazaki J. (2013). The effects of 2% rebamipide ophthalmic solution on the tear functions and ocular surface of the superoxide dismutase-1 (sod1) knockout mice. Invest. Ophthalmol. Vis. Sci..

[22] Kojima T., Simsek C., Igarashi A., Aoki K., Higa K., Shimizu T., Dogru M., Tsubota K., Shimazaki J. (2018). The Role of 2% Rebamipide Eye Drops Related to Conjunctival Differentiation in Superoxide Dismutase-1 (Sod1) Knockout Mice. Invest. Ophthalmol. Vis. Sci..

[23] Condon P.I., McEwen C.G., Wright M., Mackintosh G., Prescott R.J., McDonald C. (1999). Double blind, randomised, placebo controlled, crossover, multicentre study to determine the efficacy of a 0.1% (*w/v*) sodium hyaluronate solution (Fermavisc) in the treatment of dry eye syndrome. Br. J. Ophthalmol..

[24] Aragona P., Di Stefano G., Ferreri F., Spinella R., Stilo A. (2002). Sodium hyaluronate eye drops of different osmolarity for the treatment of dry eye in Sjogren’s syndrome patients. Br. J. Ophthalmol..

[25] Kinoshita S., Oshiden K., Awamura S., Suzuki H., Nakamichi N., Yokoi N., Rebamipide Ophthalmic Suspension Phase 3 Study Group (2013). A randomized, multicenter phase 3 study comparing 2% rebamipide (OPC-12759) with 0.1% sodium hyaluronate in the treatment of dry eye. Ophthalmology.

[26] Urashima H., Takeji Y., Okamoto T., Fujisawa S., Shinohara H. (2012). Rebamipide increases mucin-like substance contents and periodic acid Schiff reagent-positive cells density in normal rabbits. J. Ocul. Pharmacol. Ther..

[27] Alhatem A., Cavalcanti B., Hamrah P. (2012). In vivo confocal microscopy in dry eye disease and related conditions. Semin Ophthalmol..

[28] Villani E., Baudouin C., Efron N., Hamrah P., Kojima T., Patel S.V., Pflugfelder S.C., Zhivov A., Dogru M. (2014). In vivo confocal microscopy of the ocular surface: from bench to bedside. Curr. Eye Res..

[29] Villani E., Magnani F., Viola F., Santaniello A., Scorza R., Nucci P., Ratiglia R. (2013). In vivo confocal evaluation of the ocular surface morpho-functional unit in dry eye. Optom. Vis. Sci..

[30] Benitez-Del-Castillo J.M., Acosta M.C., Wassfi M.A., Diaz-Valle D., Gegundez J.A., Fernandez C., Garcia-Sanchez J. (2007). Relation between corneal innervation with confocal microscopy and corneal sensitivity with noncontact esthesiometry in patients with dry eye. Invest. Ophthalmol. Vis. Sci..

[31] Labbe A., Alalwani H., Van Went C., Brasnu E., Georgescu D., Baudouin C. (2012). The relationship between subbasal nerve morphology and corneal sensation in ocular surface disease. Invest. Ophthalmol. Vis. Sci..

[32] Simsek C., Kojima T., Nagata T., Dogru M., Tsubota K. (2019). Changes in Murine Subbasal Corneal Nerves After Scopolamine-Induced Dry Eye Stress Exposure. Invest. Ophthalmol. Vis. Sci..

[33] Zhang X.C., Kainz V., Burstein R., Levy D. (2011). Tumor necrosis factor-alpha induces sensitization of meningeal nociceptors mediated via local COX and p38 MAP kinase actions. Pain.

[34] Hosal B.M., Ornek N., Zilelioglu G., Elhan A.H. (2005). Morphology of corneal nerves and corneal sensation in dry eye: a preliminary study. Eye.

[35] Tuominen I.S., Konttinen Y.T., Vesaluoma M.H., Moilanen J.A., Helinto M., Tervo T.M. (2003). Corneal innervation and morphology in primary Sjogren’s syndrome. Invest. Ophthalmol. Vis. Sci..

[36] Zhang M., Chen J., Luo L., Xiao Q., Sun M., Liu Z. (2005). Altered corneal nerves in aqueous tear deficiency viewed by in vivo confocal microscopy. Cornea.

[37] Villani E., Galimberti D., Viola F., Mapelli C., Del Papa N., Ratiglia R. (2008). Corneal involvement in rheumatoid arthritis: An in vivo confocal study. Invest. Ophthalmol. Vis. Sci..

[38] Villani E., Garoli E., Termine V., Pichi F., Ratiglia R., Nucci P. (2015). Corneal Confocal Microscopy in Dry Eye Treated with Corticosteroids. Optom. Vis. Sci..

[39] Arakaki R., Eguchi H., Yamada A., Kudo Y., Iwasa A., Enkhmaa T., Hotta F., Mitamura-Aizawa S., Mitamura Y., Hayashi Y. (2014). Anti-inflammatory effects of rebamipide eyedrop administration on ocular lesions in a murine model of primary Sjogren’s syndrome. PLoS ONE.

[40] Ueta M., Sotozono C., Yokoi N., Kinoshita S. (2013). Rebamipide suppresses PolyI:C-stimulated cytokine production in human conjunctival epithelial cells. J. Ocul. Pharmacol. Ther..

[41] Bron A.J., de Paiva C.S., Chauhan S.K., Bonini S., Gabison E.E., Jain S., Knop E., Markoulli M., Ogawa Y., Perez V. (2017). TFOS DEWS II pathophysiology report. Ocul. Surf..

[42] Basbaum A.I., Bautista D.M., Scherrer G., Julius D. (2009). Cellular and molecular mechanisms of pain. Cell.

[43] Choi E.Y., Kang H.G., Lee C.H., Yeo A., Noh H.M., Gu N., Kim M.J., Song J.S., Kim H.C., Lee H.K. (2017). Langerhans cells prevent subbasal nerve damage and upregulate neurotrophic factors in dry eye disease. PLoS ONE.

[44] Yamazaki R., Yamazoe K., Yoshida S., Hatou S., Inagaki E., Okano H., Tsubota K., Shimmura S. (2017). The Semaphorin 3A inhibitor SM-345431 preserves corneal nerve and epithelial integrity in a murine dry eye model. Sci. Rep..

[45] Stepp M.A., Pal-Ghosh S., Tadvalkar G., Williams A., Pflugfelder S.C., de Paiva C.S. (2018). Reduced intraepithelial corneal nerve density and sensitivity accompany desiccating stress and aging in C57BL/6 mice. Exp. Eye Res..

[46] Miwa H., Osada T., Nagahara A., Ohkusa T., Hojo M., Tomita T., Hori K., Matsumoto T., Sato N. (2006). Effect of a gastro-protective agent, rebamipide, on symptom improvement in patients with functional dyspepsia: A double-blind placebo-controlled study in Japan. J. Gastroenterol. Hepatol..

[47] Naito Y., Yoshikawa T., Tanigawa T., Sakurai K., Yamasaki K., Uchida M., Kondo M. (1995). Hydroxyl radical scavenging by rebamipide and related compounds: Electron paramagnetic resonance study. Free Radic Biol. Med..

[48] Moon S.J., Woo Y.J., Jeong J.H., Park M.K., Oh H.J., Park J.S., Kim E.K., Cho M.L., Park S.H., Kim H.Y. (2012). Rebamipide attenuates pain severity and cartilage degeneration in a rat model of osteoarthritis by downregulating oxidative damage and catabolic activity in chondrocytes. Osteoarthritis Cartilage.

[49] Galor A., Moein H.R., Lee C., Rodriguez A., Felix E.R., Sarantopoulos K.D., Levitt R.C. (2018). Neuropathic pain and dry eye. Ocul. Surf..

[50] Faul F., Erdfelder E., Lang A.G., Buchner A. (2007). G*Power 3: A flexible statistical power analysis program for the social, behavioral, and biomedical sciences. Behav. Res. Methods.

[51] Shimmura S., Ono M., Shinozaki K., Toda I., Takamura E., Mashima Y., Tsubota K. (1995). Sodium hyaluronate eyedrops in the treatment of dry eyes. Br. J. Ophthalmol..

[52] Oliveira-Soto L., Efron N. (2001). Morphology of corneal nerves using confocal microscopy. Cornea.

